# Cell fusion as a driver of metastasis: re-evaluating an old hypothesis in the age of cancer heterogeneity

**DOI:** 10.3389/fimmu.2025.1524781

**Published:** 2025-02-04

**Authors:** Eduardo López-Collazo, Laura Hurtado-Navarro

**Affiliations:** ^1^ The Innate Immune Response Group, IdiPAZ, La Paz University Hospital, Madrid, Spain; ^2^ Tumour Immunology Laboratory, IdiPAZ, La Paz University Hospital, Madrid, Spain; ^3^ CIBER of Respiratory Diseases (CIBERES), Madrid, Spain; ^4^ UNIE University, Madrid, Spain

**Keywords:** metastasis, cell fusion, macrophages, cancer cell hybrid, tumour hybrid cell, cancer recurrence, oncogenic resistance

## Abstract

Numerous studies have investigated the molecular mechanisms and signalling pathways underlying cancer metastasis, as there is still no effective treatment for this terminal stage of the disease. However, the exact processes that enable primary cancer cells to acquire a metastatic phenotype remain unclear. Increasing attention has been focused on the fusion of cancer cells with myeloid cells, a phenomenon that may result in hybrid cells, so-called Tumour Hybrid Cells (THCs), with enhanced migratory, angiogenic, immune evasion, colonisation, and metastatic properties. This process has been shown to potentially drive tumour progression, drug resistance, and cancer recurrence. In this review, we explore the potential mechanisms that govern cancer cell fusion, the molecular mediators involved, the metastatic characteristics acquired by fusion-derived hybrids, and their clinical significance in human cancer. Additionally, we discuss emerging pharmacological strategies aimed at targeting fusogenic molecules as a means to prevent metastatic dissemination.

## Historical context and theoretical framework

Metastasis is the process by which cancer cells spread beyond the site of the primary niche to other parts of the organism, generating secondary tumours that can compromise the affected tissues’ vital functions. This process implies that freed cancer cells, known as Circulating Tumour Cells (CTCs), leave their primary site, enter, circulate and survive in the bloodstream and lymphatic vessels, withstand their pressure, extravasate, and are finally able to reach and colonize secondary niches escaping the “combat” with immune cells (1, 2).

Woefully, this is the final stage for a large proportion of cancer patients, accounting for more than 90% of cancer-related deaths (3). Despite its high prevalence, metastasis is regarded as an extraordinarily inefficient process as it encompasses a series of complex challenges which include the removal of intravasated cancer cells by hemodynamic forces, immune clearance, metabolic stress, apoptosis, and anoikis. Each barrier significantly reduces the likelihood of a cancer cell completing the metastatic process. In fact, few clinically relevant metastases are formed compared to the number of freed cells from tumours in circulation (4).

One of the earliest and most renowned theories used to explain the metastatic spread was the “Seed and Soil” theory, proposed by Stephen Paget in 1889 (5). Paget stated that the locations where distant metastasis arise are not elected by chance. Rather, they are selected because of the existence of tumour cells with metastatic potential (the “seeds”) and tissues or organs (the “soils”) with the appropriate environment for the colonization and proliferation of the aforementioned “seeds”, being these two elements compatible among themselves (6). The underlying mechanisms that may explain this phenomenon, known as organotropism or organ-specific metastasis, include the patterns of blood circulation that determine the accessibility of secondary organs, the intrinsic genetic and epigenetic factors associated with the tumour, as well as the organ-specific microenvironment that establishes the formation of supportive premetastatic niches (7, 8).

This theory, while influential, oversimplifies the complex metastatic process by focusing solely on cancer cells and target organs, neglecting factors like mechanical influences, immune responses, cancer cell plasticity, and the pre-metastatic niche. Additionally, it primarily addresses late-stage metastasis and lacks direct therapeutic applications.

In contrast to the “Seed and Soil” theory, James Ewing proposed in 1928 that the circulatory patterns connecting the primary tumour and the disseminated secondary organs are enough to explain organ-specific metastasis (9). In other words, mechanical factors resulting from the anatomical structure of the vascular system are sufficient to explain the metastatic dissemination to concrete secondary sites. Notably, this concept has been shown to align with the observed pattern of metastatic spread.

The previously cited theories merely indicate the expected locations of metastases. Nonetheless, they do not describe the whole metastatic cascade process; in this regard, the most widely accepted explanation for the alterations observed in disseminated cancer cells is the epithelial-to-mesenchymal transition (EMT).

EMT is the process through which cells lose their epithelial characteristics and acquire a mesenchymal phenotype and increased plasticity. This transition has been linked to several tumour-related functions, including tumour initiation, malignant progression, enhanced cancer cell migration, increased mobility, metastatic potential, and resistance to therapy (10).

During EMT, cells undergo a spectrum of transitional changes in gene expression and post-translational regulation mechanisms that lead to the repression of the epithelial characteristics and the acquisition of mesenchymal features. This results in the presence of multiple cell populations, each expressing varying levels of both epithelial and mesenchymal markers, along with intermediate morphological, transcriptional and epigenetic profiles, oscillating between the two phenotypes (11).

The main defining feature of EMT is the loss of the epithelial marker E-cadherin and the gain of Vimentin mesenchymal marker. Nevertheless, several other features including cytokeratins, CD106, CD61, CD51 and N-cadherin are also involved (12). Tumour cells co-expressing both epithelial and mesenchymal markers, indicating the occurrence of EMT, have been identified in various human primary cancers, including breast, colorectal, head and neck squamous cell carcinoma, lung, and esophageal cancers, among others (12–15). This phenotype is associated with enhanced invasive and migratory abilities, increased survival in suspension, and more efficient colonization at secondary sites. In addition, these characteristics facilitate metastatic spread and underscore the critical role of EMT in this process (10, 16–18).

Despite the extensive scientific evidence highlighting the importance of EMT in carcinogenesis, certain studies have raised critical questions about its functional relevance in specific cancer types. In particular, there remains active debate over whether EMT primarily facilitates the early stages of the metastatic cascade or if the same cells also play a key role in driving metastatic outgrowth at distant sites (19, 20). In fact, scientific evidence supports the notion that EMT contributes to metastatic spread; however, it is not a prerequisite for metastasis in certain types of cancer (21, 22).

Remarkably, the EMT theory of metastasis, while influential, faces several criticisms. It struggles to explain why metastatic lesions often retain epithelial traits, relies on reversible EMT and mesenchymal-to-epithelial transition (MET) concepts, and has been challenged by *in vivo* studies showing EMT may not be essential for metastasis (21–23). Additionally, the theory may oversimplify the metastatic process, and findings from cell culture experiments may not fully reflect *in vivo* complexity (24). Furthermore, difficulties in tracking EMT events, incomplete suppression in genetic studies, and evidence of early cancer cell dissemination challenge the idea that EMT is a prerequisite for metastasis (25).

Other mechanisms, including trans-differentiation—the conversion of a differentiated cell into another specialised cell lineage—and dedifferentiation, where a differentiated cell reverts to an undifferentiated state within its own lineage, have received comparatively less attention in the study of metastasis. Nevertheless, emerging evidence highlights their critical role in tumour plasticity, enabling transitions between distinct cellular states that significantly contribute to tumour initiation, progression, metastasis, and therapy resistance (26–28).

In efforts to unravel the mechanisms underlying metastasis, one event that has intermittently gained attention in the field is cell fusion. This phenomenon is well-documented in several fundamental biological processes, including fertilisation, mesenchymal cell differentiation, development, regeneration, and wound healing (29). It contributes to the genotypic and phenotypic diversity of the resulting cells in comparison with the original ones (30).

Besides their regulated physiological role, fusion episodes have also been detected in various pathological conditions, including cancer (31–34). Moreover, specific traits of macrophages have been found in metastatic cells of different types of cancer, suggesting that bone marrow derived cells (BMDC)-tumour cell hybrids or Tumour Hybrid Cells (THCs) might participate in cancer initiation and metastasis (35–37). Moreover, the investigation into the relationship between tumour cell hybrids and CTCs has garnered significant attention, as it could enhance our understanding of metastasis, enable its early detection, and identify potential therapeutic targets.

Nonetheless, the “Cancer Cell Fusion” is not a novel theory. As early as the beginning of 20^th^ century, observations suggested that spontaneous fusion between leukocytes and cancer cells could be a potential source of aneuploidy, a condition that may contribute to the development of metastatic cells (38). Later on, Mekler and Goldenberg independently gave experimental evidence that supported the fusion model for metastasis (39, 40).

Since then, the “Cancer Cell Fusion” theory has gained recognition as a potential additional explanation for tumour progression (41–43). This is because fusion represents a non-mutational mechanism that might explain the aberrant gene expression profiles found on metastatic cells (42). Moreover, the genes predominantly expressed during EMT closely resemble those associated with migratory bone marrow-derived cells, suggesting the possibility that EMT may result from the fusion of cancer cells with BMDCs. In addition, the altered gene expression observed in hybrid cells –resulting from heterotypic nuclear fusion and the co-expression of both genomes – could also explain their enhanced stemness properties, augmented migration and invasion ability, drug resistance, and other metastatic hallmarks (30, 31, 44).

In fact, the “Cancer Cell Fusion” theory may explain rapid metastatic trait acquisition in cancer without relying on genetic mutations. It accounts for tumour heterogeneity, organ-specific metastasis, EMT, and immune evasion. Supported by *in vitro* and *in vivo* evidence, it offers insight into cancer progression and explains post-transplant cancers where donor genes appear in recipient cells.

Given the aforementioned information, further scientific characterization of the molecular mechanisms driving fusion events may improve the current knowledge about the metastatic dissemination process, leading to the identification of novel therapeutic approaches that could hamper this final stage of cancer progression.

## Genetic evidence of cancer cell fusion *in vivo*

Increasing scientific evidence supports that cell fusion might play an essential role in cancer biology and the development of metastasis, as it has been mentioned before (36, 44–52). Cancer cell fusion as a mechanism of tumour progression has been described in *in vitro* cultures and animal models in several published reports, even stating the possible relationship between cell fusion and metastatic spread.

Nevertheless, the limited presence of hybrids in human cancer tissues is primarily due to technical challenges related to the direct detection and tracing of the parental cell lineages involved in the fusion process (44).

One of the most convincing demonstrations of BMDC-tumour cell fusion in humans was the detection of tumour-associated osteoclasts - multi-nucleated cells of monocytic origin - that contained transcriptionally active malignant nuclei along with normal nuclei in myeloma patients (53). The contribution of myeloma B-cell nuclei within the whole osteoclast population was greater than 30%, suggesting a high rate of osteoclast-myeloma cell fusion. The formation of such hybrids was verified in myeloma cell-osteoclast co-cultures (53, 54).

Likewise, additional genetic evidence for myeloid-cancer hybrid cells was collected from two patients that developed renal cell carcinoma (RCC) after receiving an allogeneic hematopoietic stem cell (HSC) transplant.

The first case was a paediatric patient who developed RCC with metastasis after receiving an HSC transplant from his cancer-free brother. The donor and recipient blood-group genotypes were O^+^ and the donor bone marrow transplant was A^+^. Therefore, any blood cells in the tumour will be of donor genotype. After the isolation of cells from a metastasized lymph node, tumour DNA amplification and histological sections were used to compare the tumour and donor’s samples. They found that carcinoma cells contained the donor-specific A allele, indicating that BMDCs were somehow incorporated into the metastatic tissue, potentially through fusion with pre-existing tumour cells (55).

The second patient was an adult female who developed a primary papillary RCC, two years after a male-to-female HSC transplant from her cancer-free son. The RCC cells’ karyotype showed that some of them presented a common genomic abnormality observed in this type of cancer, namely trisomy in chromosome 17. The results of the FISH assays demonstrated that approximately 1% of the malignant cells containing trisomy 17 also exhibited the presence of the donor Y chromosome. As in the previous case, this evidence highlights possible fusion events between the received BMDCs and a pre-existing malignancy (34).

In addition to these two cases, there are other reports that claim the presence of Y chromosome-containing cancer cells in three female patients, two with colorectal adenoma and one with squamous cell lung cancer, who had previously received a sex-mismatch HSC transplant (56).

At the molecular level, current research has demonstrated that tumour-BMDC fusions, as macrophage-melanoma hybrids isolated *in vitro*, exhibit gene expression patterns linked to a migratory phenotype, increased survival, metastasis and poor outcome. This is the case for *SPARC* (57, 58) (a key component of wound healing and tissue repair), *MCR1* (59, 60) (a regulator of proliferation in melanoma progression) and cell surface expression of *LAMP1* (61). This elevated expression of key molecules in hybrid cells likely results from the fusion of cancer cells with migrating BMDCs, leading to the co-expression of imprinted genes from both fusion partners.

Although host cell-cancer cell fusion, including fusion with BMDCs, has been observed in humans (**Table 1**), there is still much to be discovered. The precise mechanism by which heterotypic cell fusion occurs in tumours, as well as the process by which a fusion partner is selected, are areas of ongoing research. To advance understanding in this area, it is essential to identify the specific cellular population involved in the fusion process, pinpoint potential markers that could aid in the identification of hybrid cells and further characterize the mechanisms diving cell fusion in this context.

**Table 1:** Presence of BMDCs – Cancer cell hybrids in human samples.

Cancer Type	Normal Cell	Sample Type	Reference
Multiple myeloma	Osteoclasts	Primary tumour biopsy	(53) (54)
Renal cell carcinoma	BMDCs BMDCs BMDCs Macrophages	Lymph node metastasis Primary tumour biopsy Primary tumour biopsy Liquid biopsy (CTCs)	(55) (34) (36) (62)
Melanoma	BMDCs BMDCs BMDCs Macrophages	Liquid biopsy (CTCs) Brain metastasis Lymph node metastasis Liquid biopsy (CTCs)	(63) (33) (32) (64)
Breast cancer	Macrophages Macrophages Macrophages Macrophages Macrophages	Primary tumour biopsy Liquid biopsy (CTCs) Liquid biopsy (CTCs) Liquid biopsy (CTCs) Liquid biopsy (CTCs)	(65) (66) (67) (62) (68)
Squamous cell lung cancer	Macrophages	Liquid biopsy (CTCs)	(62)
Colorectal cancer	Macrophages BMDCs Macrophages Macrophages	Primary tumour biopsy Liquid biopsy (CTCs) Liquid biopsy (CTCs) Liver and lung metastasis	(69) (63) (70) (71)
Prostate cancer	Macrophages Macrophages	Liquid biopsy (CTCs) Liquid biopsy (CTCs)	(70) (62)
Lung cancer	Macrophages BMDCs Monocytes	Liquid biopsy (CTCs) Primary tumour biopsy Primary tumour biopsy Liquid biopsy (CTCs)	(62) (36) (47)
Oesophageal cancer	Macrophages	Liquid biopsy (CTCs)	(62)
Pancreatic cancer	BMDCs BMDCS Macrophages Macrophages Macrophages	Primary tumour biopsy Liquid biopsy (CTCs) Liquid biopsy (CTCs) Liquid biopsy (CTCs) Liquid biopsy (CTCs) Liquid biopsy (CTCs)	(36) (63) (70) (62) (37)
Ovarian carcinoma	BMDCs	Ascites	(72)
Head and neck squamous cell carcinoma	BMDCs	Primary tumour biopsy	(36)

## Macrophage contribution to tumorigenesis: evidence of macrophage characteristics in human tumour samples

Macrophages are a versatile population of myeloid-lineage cells, primary originating as monocytes from hematopoietic stem cells in the bone marrow. These monocytes continuously circulate in the bloodstream, migrating to target tissues where, under inflammatory conditions, they differentiate into tissue-resident macrophages. As phagocytic cells, macrophages play a diverse and crucial role in development, maintaining tissue homeostasis, and regulating inflammatory and immune responses (73, 74).

These cells exhibit remarkable plasticity, undergoing “polarization” in response to environmental stimuli like cytokines and signalling mediators (75). According to the binary polarization concept, classically activated macrophages (M1) adopt a pro-inflammatory phenotype, releasing cytokines (e.g., IL-12, IL-23, TNF-α) and reactive oxygen species, essential for pathogen defence and cancer cell elimination. M1 activation is triggered by LPS, GM-CSF, and Th1 cytokines (IFN-γ, TNF-α). Conversely, alternatively activated macrophages (M2) are induced by Th2 cytokines (IL-4, IL-13), CSF-1, and TGF-β, promoting anti-inflammatory responses, inflammation resolution, angiogenesis, and tissue repair (76–78).

Focusing on macrophage populations related to the process of tumorigenesis, it has been described that tumour-associated macrophages (TAMs) are recruited into the tumour microenvironment (TME) by cancer cells, inflammatory cytokines and growth factors, such as chemotactic chemokine (CCL2) and CSF-1/M-CSF, among other molecules (79, 80).

The majority of TAMs within TME correspond to M2 polarized macrophages, which indeed display pro-tumorigenic effects, in contrast to anti-tumour properties of M1 macrophages (81). The M2 TAMs have been shown to contribute to malignant transformation, cancer cell survival and proliferation, tumour growth, angiogenesis, invasion and metastasis, while simultaneously suppressing the immune response towards cancer cells and to the standard-of-care therapeutics, such as chemotherapy and radiotherapy (82, 83).

In addition to the direct association between M2 TAMs and tumour cells, they have critical interactions with other cell populations within TME that are associated with disease progression. These include Th2 cells, cancer-associated fibroblasts (CAFs), regulatory T cells (Tregs), myeloid-derived suppressor cells and others. Additionally, negative cross-talk occurs between M2 macrophages and tumour-suppressing cells, such as cytotoxic T cells and natural killer (NK) cells (77). Due to their pro-tumorigenic actions, an increased density of TAMs in the TME is generally linked to poor prognosis in most human cancers (77, 84).

A wide variety of markers are used to identify macrophages in both clinical and research fields: CD14 (co-receptor to toll-like receptor 4, TLR4, for detection of bacterial lipopolysaccharide, LPS), CD68 (involved in antigen processing and caption of low-density lipoprotein, LDL), CD163 (scavenger receptor for the haptoglobin-haemoglobin complex), CD206 (mannose receptor), DAP12 (macrophage fusion protein) and MAC387 (calprotectin molecule), among others (65, 69).

As some evidences of the hybridization phenomenon between macrophages and cancer cells *in vivo*, the spontaneous fusion in co-cultures of human MCF-7 breast cancer cells with M2 macrophages, activated from monocytes obtained from blood circulation, was reported in different publications (55, 56). Subsequent studies similarly reported that highly oxidised M2-polarised monocytes exhibit significantly higher rates of fusion with human lung cancer cells *in vitro* compared to M1 monocytes (47). Further confirmation is retrieved from the analysis of melanoma – macrophage hybrids isolated from CTCs populations from peripheral blood samples of patients. When cultured, they expressed specific M2 polarization markers (CD163, CD204, CD206) (35), providing evidence of M2 macrophages role as fusogenic partners of different types of cancer cells both *in vitro* and *in vivo*, generating THCs (**Table 1**).

Overall, evidence suggests that the presence of macrophage characteristic in cancer cells is linked to a worse poor prognosis, including a lower recurrence-free survival, more advanced tumour histology and increased metastasis. The presence of these markers in cancer cells, along with the *in vivo* evidence of cell fusion discussed earlier, could be explained by the “Cancer Cell Fusion” theory. This model posits that malignant cells are able to fuse with BMDCs, especially monocytes and macrophages, resulting in hybrid cells with enhanced tumorigenic, migratory abilities and metastatic potential (42).

## Mechanism of cancer cell fusion and molecular mediators

The precise mechanisms and molecules driving cell fusion remain an appealing yet underexplored area of research. Additionally, the immune system basis of the process that favours this process during the tumour cascade remains to be elucidated. The use of model organisms has enabled the characterisation of the three fundamental steps thought to be essential for cell-cell fusion at least in non-pathological conditions.

These stages include: i) Competence, involving the differentiation of cells into fusion-competent forms through recognition of extracellular signals, cell polarization, migration, morphological changes and surface expression of specific molecules. ii) Commitment, where cell-cell adhesion extends the recognition and polarization process, leading to the activation of fusogenic machinery. iii) Cell-cell fusion, which involves the merging of plasma membranes, mixing of cytoplasmatic contents, the rearrangement and fusion of chromosomes from the previously discrete nuclei, and subsequent signalling and developmental changes (85).

With this in mind, it could be hypothesised that molecular triggers capable of initiating and completing the fusion process likely exist both in the cellular environment and on the surface of the involved cells.

Despite the paucity of specific research in the field of cell fusion, microtubules are a potential candidate for investigation, given their indirect influence on cancer and immune cell interactions, including processes such as cell division, migration, intracellular transport, and EMT transition (86). Indeed, microtubules and their associated proteins (MAPs) play a critical role in cytoskeletal remodeling, a hallmark of tumour cell plasticity and hybrid cell formation (87). Furthermore, alterations in microtubule stability and the expression of specific tubulin isotypes have been observed across various cancers (86). These changes are implicated in the transport of functional molecules that contribute to the formation of the CSC niche (88). Recent studies also indicate that microtubule-targeting agents (MTAs), such as paclitaxel, may induce mitotic cell death while simultaneously increasing tumour-infiltrating immune cells (89, 90). In view of these observations, it is reasonable to hypothesize that microtubules might play an indirect role in the formation of hybrid cells through microtubule-mediated communication between cancer cells and immune cells.

According to the fusion of cell membranes, it is mediated by specialised cellular proteins known as “fusogens”, which facilitate the close proximity of membrane bilayers of cells, enabling the rearrangement of proteins within the membrane (31). To date, several fusogens have been identified. For example, syncytins are a well-characterized family of mammalian fusogen from endogenous retroviruses, primarily involved in the formation of syncytial trophoblasts during placentation (31). Abnormal expression of these proteins has been linked to cancer cell fusion and the development and progression of various tumours (91–93). However, significant knowledge gaps remain regarding human cell fusogens, particularly, those contributing to fusion in cancer cells, although promising candidates are emerging.

Focusing on macrophages, these cells play a fundamental role in the formation of both osteoclasts and giant cells through homotypic fusion, a process essential for bone maintenance and immune response regulation. Achieving these functions requires the expression of various fusion-related genes and pathways within macrophages.

One key player in this process is the macrophage fusion receptor (MFR), also known as signal-regulatory protein α (SIRPα), a plasma membrane protein belonging to the immunoglobulin superfamily, which is expressed by both myeloid cells and neurons. Its ligand, CD47, is also a member of the immunoglobulin superfamily. Its expression is ubiquitous across cell types, with particularly high levels observed on the surfaces of tumours and CTCs. During macrophage homotypic fusion, SIRPα expression is transiently induced at the onset, while CD47 levels remain constant throughout the process (94, 95).

In addition to the SIRPα-CD47 interaction, other molecules are involved in the regulation of macrophage fusion. This is the case of CD44, a transmembrane glycoprotein found in various cell types, including embryonic stem cells and cells of connective tissue and bone marrow. CD44 plays a pivotal role in this process. Its primary ligand, hyaluronic acid (HA), is abundant in the extracellular matrix. CD44 is frequently upregulated in cancer stem cells (CSCs), making it a molecular marker for their identification (96). Interestingly, CD44 levels are transiently increased during the early stages of macrophage fusion, suggesting an active role in this process (94). Beyond macrophage fusion, overexpression of CD44 and CD47 in colorectal cancer has been associated with advanced tumour progression, distant metastasis, and a reduced disease-free survival rate. Moreover, stage IV recurrent tumours following treatment exhibit high levels of these markers and an EMT phenotype (97, 98).

CD47, in addition to its role in fusion, acts as a phagocytosis-suppressing signal, helping tumour cells and CTCs evade recognition and phagocytic clearance by the immune system. This ability to escape immune surveillance increases tumour aggressiveness and metastatic potential (99, 100). As a result, considerable efforts have been made to develop immunotherapies targeting the CD47-SIRPα axis, with promising results. Anti-CD47 antibody therapy has demonstrated potent anti-tumour activity by enhancing tumour cell phagocytosis by macrophages in various cancer types, including leukaemia, non-Hodgkin lymphoma, cholangiocarcinoma, colon cancer, glioma, and non-small cell lung cancer. This therapy has also been shown to inhibit cancer cell proliferation and metastasis in mouse models, thereby increasing survival and reducing tumour aggressiveness (101, 102).

Currently, several CD47-targeting antibodies, either alone or in combination with chimeric antigen receptor (CAR) T cells, are being tested in clinical trials, with varying degrees of success depending on the cancer type (103, 104). From the perspective of the “Cancer Cell Fusion” theory, a possible explanation for the favourable outcomes of CD47-SIRPα blockade is that CD47 on cancer cells may act as a trigger for macrophage-cancer cell fusion, mediated by the SIRPα marker on myeloid cells. This fusion could produce THCs with enhanced immune evasion and metastatic capabilities. Consequently, anti-CD47 treatments may reduce fusion rates and tumour immune evasion, presenting a novel therapeutic avenue. These findings pave the way for further research into metastasis initiation and the development of strategies to prevent cancer dissemination. Exploring other factors and mediators potentially involved in the phenomenon we are analysing; lipids have been identified as molecules of interest in this context.

Lipid metabolism is a fundamental pathway in tumorigenesis, providing the energy requirements of cancer cells (105–107). CD36, a transmembrane glycoprotein that is expressed in a variety of cell types including tumour cells in malignancies (108), is involved in lipid homeostasis, angiogenesis, immune response, and cell adhesion, primarily through the uptake of fatty acids. However, it plays a significant contribution in the context of metastasis, where it promotes tumour progression and dissemination (109, 110). Along these lines, different studies showed that high expression of CD36 in oral squamous cell carcinoma (OSCC) metastasis-initiating subpopulation (111) and breast cancer cells (112) correlates with an increased dissemination ability and therapeutic resistance, respectively. Its blockade, using antibody-mediated abrogation or its deletion, exerts an inhibition of tumour dissemination and growth (111, 112).

Examining cell fusion processes in the context of metastasis, Aguirre et al., reported that CD36 fusogenic activity plays a role in mediating lung cancer cell-monocyte spontaneous fusion. This event results in the formation of THCs, which exhibit enhanced proliferation, migration, immune avoidance and *in vivo* metastatic behaviour (47). In fact, CD36 overexpression on H460 lung cancer cells increased the ratio of THCs formation, whereas no significant increase was reported when monocytes overexpressed CD36. Additionally, RNA interference (RNAi)-mediated reduction of CD36 expression in H460 cells significantly decreased the occurrence of hybridisation. Furthermore, the THC-specific cell surface signature (CD36^+^CD14^+^PANK^+^) enables the identification of these cells in matched primary tumour tissues and metastases, as well as in the bloodstream of patients with lung cancer, acting as a biomarker (47).

Overall, CD36 appears to serve a dual function in metastasis. Firstly, it regulates the uptake of fatty acids, providing the necessary energy for cellular processes (111). Secondly, it acts as a fusogen, facilitating the fusion-mediated generation of metastatic cells. Further research is essential to investigate CD36’s role in the spread of other cancer types, which could confirm this hypothesis and pave the way for future clinical applications of anti-metastatic therapies targeting this fusogen. Eventually, inflammatory cytokines, such as TNF-α, have been shown to induce cell fusions events, highlighting the importance of this process in tumour progression (46, 113).

An important point to note is that the process of fusion between macrophages and tumour cells is favoured when the latter are stem cells (114, 115). In this line, the cell fusion process is not only limited to cancer stem cells, but also to other stem cells such as human adipose-derived mesenchymal stem cells (hADMSCs), which have been shown to fuse with monocytes *ex vivo*, generating new hybrid cellular entities defined as foam hybrid cells (FHCs) (116).

**Figure 1:**
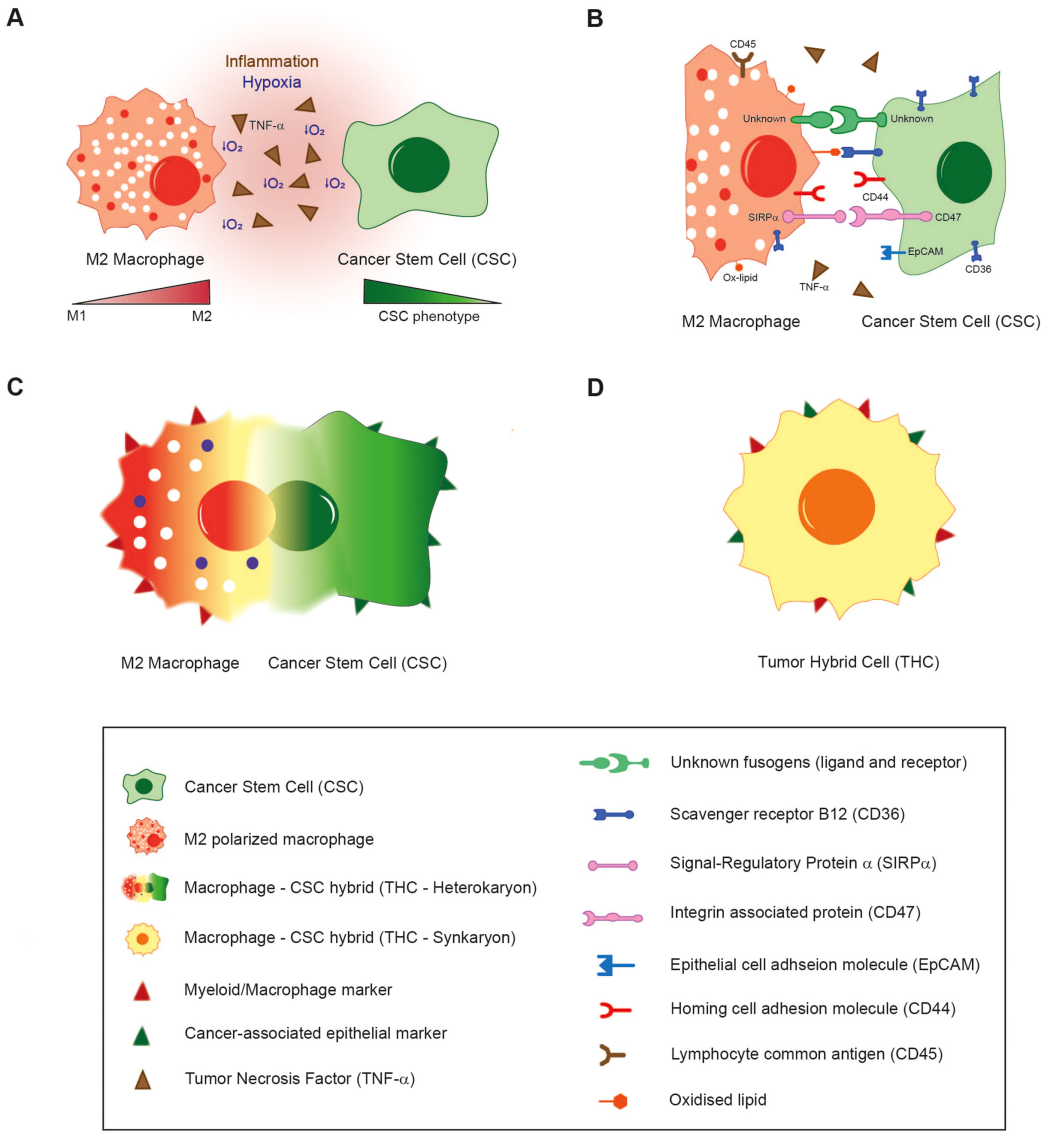
The proposed fusion mechanism of macrophages with Cancer Stem Cells. **(A)** Inflammatory conditions, hypoxia and the activation of wound healing/tissue regeneration response in the TME induce the fusion of M2-polarized macrophages with CSCs. **(B)** Several environmental and cellular-surface molecular mediators are under scrutiny for their putative role in hybridization. **(C)** After first interaction through receptors, plasma membranes of both cell types are supposed to merge, giving rise to a bi- or multinucleated heterokaryon fusion-derived cell with an enlarged cytoplasm. **(D)** Hybridization is completed when nuclei are merged (heterokaryon-to-synkaryon transition), originating a synkaryon THC which expresses both epithelial and myeloid markers, as well as other novel traits.

In light of the aforementioned considerations, it has been demonstrated that inflammatory conditions, hypoxia, necrosis and the wound healing response, all of which are present within the TME, participate in the induction of hybridization between tumour cells and surrounding populations (**Figure 1A**). Furthermore, it is reasonable to propose that cellular fusion is a stepwise process governed by strict regulatory mechanisms. During a pre-hybrid preparation phase, somatic or cancer cells transition into a pro-fusogenic state, requiring modifications such as cytoskeletal reorganisation. Even though several molecules and factors are in the spotlight (**Figure 1B**), like syncytins and phosphatidylserine, the vast majority of mediators are still unknown, and the precise fusion machinery that initiates this process has yet to be fully elucidated. In this regard, a deeper characterization is necessary to comprehend the whole process and to identify weak targets for these specific hybrid tumour subpopulations.

According to the sequence of the steps involved in fusion, the initial resultant hybrid would be constituted of two or more individual nuclei and an enlarged cytoplasm (heterokaryon cell) (**Figure 1C**). These fused cells could undergo heterokaryon to synkaryon transition, resulting in the merging and fusion of their nucleus, giving rise to a single or multinucleated cell composed of the genetic material of the parental cells involved in the hybridization (117). A consequence of these transition is the emergence of novel traits acquired through the genetic recombination of at least two nuclei. This process leads to simultaneous expression of both myeloid and cancer-related epithelial markers (**Figure 1D**). Several other phenotypic changes are also linked to cell fusion, with the most commonly reported of which is the acquisition of a metastatic behaviour (**Table 2**).

**Table 2:** BMDCs – Cancer cell hybrids’ acquired characteristics.

Phenotype	Fused cells	Reference
Enhanced migration, motility, chemotaxis and invasion	Melanoma cell – Macrophage Melanoma cell – Macrophage Melanoma cell – Macrophage Pancreatic cancer cell - Macrophage Colorectal cancer cell – Macrophage Melanoma cell – Macrophage Breast cancer cell – Macrophage Lung cancer cell – Macrophage Ovarian cancer cell - BMDCs Colorectal cancer cell – Monocyte	(118) (119) (64) (37) (36) (21) (120) (47) (72) (121)
Immune avoidance	Lung cancer cell – Macrophage Colorectal cancer cell – Monocyte	(47) (121)
Angiogenic potential	Sarcoma – Macrophage Breast cancer (CAMLs) Pancreatic cancer (CAMLs)	(122) (70) (70)
Radioresistance	Breast cancer cell – Macrophage	(123)
Chemoresistance	Breast cancer cell – Macrophage	(124)

## The timing of hybrid cell formation: immunoediting stages of cancer

The concept of cancer immunoediting has been refined over the past two decades to encompass the many facets of immune system-tumour interactions. This process consists of three phases: elimination, equilibrium and escape (125, 126). There is no definitive evidence on the timing of cell fusion, but evidence suggests it occurs at all stages of cancer immunoediting, facilitating tumour cell evolution and adaptation.

During the elimination phase, the immune system recognises and destroys transformed tumour cells. However, evidence suggests that THCs can evade immune surveillance and facilitate metastasis by upregulating immune checkpoint molecules such as CTLA-4, PD-1, and SIGLEC-5 (116, 121, 127, 128). During the equilibrium phase, tumour growth is regulated, and cellular immunogenicity is shaped by the adaptive immune system. *In vitro* co-culture models have demonstrated that some THCs, once formed, initially display a dormant phenotype, in contrast to their primary tumour counterparts (129). In the escape phase, tumour cells that have undergone immune editing proliferate without restriction. Studies indicate that tumourigenic hybrids are characterized by accelerated proliferation rates, poor prognoses, and enhanced metastatic potential (42, 52).

Collectively, these findings underscore the potential role of cell fusion in enabling tumour cells to evade immune responses, adapt to selective pressures, and drive cancer progression across all stages of immunoediting.

## Metastatic traits of hybrids acquired through fusion

Cancer is a highly heterogeneous disease, both at clinical and genomically level, with significant variation between patients, tumour types, and even within individual tumours. This diversity in tumour subpopulations is multifaceted and likely stems from a complex interplay of factors, including the origin of primary tumour and cells, the presence of genetic mutations, and histopathologic morphologies. Spontaneous mutations, selective pressures exerted by the tumour microenvironment, and the therapeutic interventions such as radiotherapy and chemotherapy all contribute to this diversity (130, 131).

The “Cancer Cell fusion” theory proposes that cell fusion plays a role in generating rapid changes within tumour hybrids, primarily through polyploidization —where a cell contains more than two sets of chromosomes— along with the transition from heterokaryon to synkaryon transition, where parental genomes could fuse into a single or more nucleus per cell. This process leads to nuclear reprogramming and epigenetic alterations, including aneuploidy (132). It suggested that tumour heterogeneity may arise from this fusion-driven mechanism, enabling THCs to rapidly acquire metastatic traits and drug resistance at a rate greater than that of random mutations (133). Understanding the fusion-acquired characteristics of hybrids is therefore critical in predicting tumour evolution (**Table 2**). Such knowledge could inform more effective therapeutic strategies, potentially controlling cancer spread and improving survival outcomes for patients.

Regarding fusion events between macrophages/monocytes and tumour cells both *in vitro* and *in vivo*, one of the most frequently reported consequences of this hybridization is increased motility, migration and invasion capabilities observed in THCs. Decades ago, Rachkovsky et al., already described that B16F10 melanoma cells – macrophages hybrids exhibited higher chemotaxis in response to fibroblast-conditioned media *in vitro*, as well as histologically-determined vascular invasion and spread to distant organs *in vivo* (118). In fact, a detailed characterization of the migratory phenotype was carried out by Ramakrishnan et al., using spontaneous hybrids between murine epithelial ovarian carcinoma (transformed GFP^+^ ID8 cells) and murine BMDCs (obtained from ascites) (72). These observations were in accordance with the enhanced migration and invasion activities of MC38 (murine colorectal cancer cells) and B16F10 (murine melanoma cells) – macrophages (murine) spontaneous hybrids (36) and polyethylene glycol (PEG)-induced N2O2 (murine breast cancer cells) – RAW264.7 (murine macrophages) hybrids (120), as later depicted.

Along these lines, Aguirre et al., described that THCs resulting from fusions between human monocytes and H460/A549 lung cancer cell lines showed *in vitro* augmented migration, invasiveness and proliferation. Furthermore, after inoculating these hybrids in mice, THCs were found to colonize distant tissues, such as lungs, lymph nodes and spleen. This demonstrated for the first time the patent *in vivo* metastatic and colonization potential of fully human-origin THCs (47). The authors also showed *in vitro* that colorectal THCs derived from the fusion of human monocytes and SW620 cell line (human colorectal cancer cells) exhibited a high rate of migration and proliferation compared to their parental populations. Moreover, the analysis of two human cohorts suggested the potential relevance of resident tissue THCs in the generation of distant metastases *in vivo* (121).

**Figure 2:**
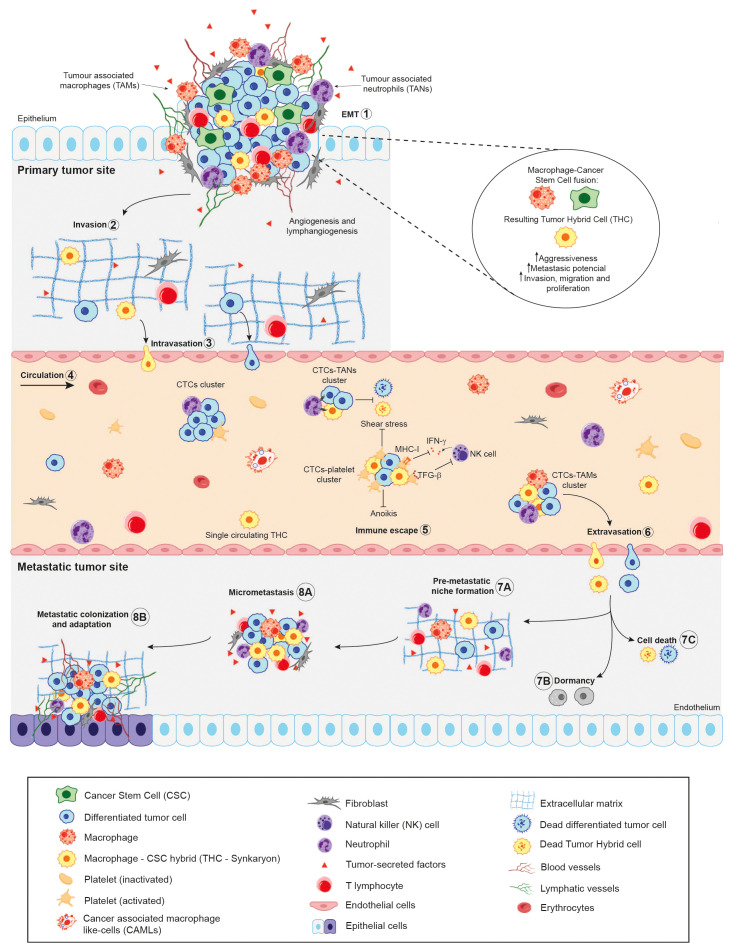
Schematic illustration of the role of Cancer Stem Cells (CSCs) in the metastatic cascade. In light of the cellular fusion hypothesis, the primary tumour will contain a combination of Tumour Hybrid Cells (THCs), resulting from the fusion of CSCs and immune cells –mainly macrophages—, CSCs and differentiated tumour cells. During metastatic dissemination, the tumour cells (1) undergo epithelial-to-mesenchymal transition (EMT), acquiring mesenchymal features from epithelial cells (10); detach from primary tumour site and (2) locally invades surrounding matrix and tissues; (3) leading to their intravasation into the bloodstream by disrupting endothelial junctions (136). This step could be performed by single cells or clusters of cells, being now called Circulating Tumour Cells (CTCs). (4) Once in circulation, the majority of CTCs and THCs succumb, either unable to endure the physical stress of shear forces or as a result of immune system assaults. (5) Within the immune escape mechanisms of CTCs and hybrid cells, they form clusters with immune cells, such as platelets, thereby impairing the cytotoxicity of Natural Killer (NK) cells by decreasing their proliferation and interferon-γ (IFN-γ) production. Additionally, by forming a platelet-rich thrombus, they act as a physical barrier for cancer cells (137–140). Furthermore, the recruitment of neutrophils, which bind to CTCs via VCAM1-mediated clustering, has been observed. These neutrophils, known as tumour-associated neutrophils (TANs), have been shown to promote tumour cell survival and proliferation (140, 141). In the context of macrophages, tumour-associated macrophages (TAMs) have been reported to stimulate epithelial-to-mesenchymal transition (EMT) in CTCs, thereby enhancing their metastatic colonisation potential (140, 142). (6) After extravasation from the bloodstream, the CTCs could seed in the microenvironment of foreign tissues, which is known as the pre-metastatic niche (7A), undergo dormancy (7B) or die (7C). Finally, cancer cells may adapt and colonise the foreign tissue microenvironment, leading to the formation of micro metastasis (8A) and secondary tumours (8B).

To disseminate throughout the body, aggressive cancer cells must detach from the primary tumour and enter the vascular or lymphatic system. During this detachment and subsequent migration, tumour cells undergo a series of cellular changes to ensure their survival. These include the disruption of cell-cell connections (mediated by molecules such as desmosomes and E-cadherin), the breakdown of extracellular matrix-cell interactions (mediated by integrins), alterations in mechanical forces, and cytoskeletal reorganisation (critical for the formation of migratory structures), among other adaptations (134, 135). As the majority of tumour cells originate from epithelial tissue and grow attached to each other and to the surrounding stroma, forming a tissue structure, the circulation is a hostile environment for them (**Figure 2**). Likewise, circulating cancer cells are continuously exposed to the immune response, *ergo* they must survive at a rate sufficient enough to seed into secondary niches and form metastases (143).

In this context, lung cancer cell–monocyte hybrids have been shown to downregulate the immune response, including perforin production and cytotoxicity, when co-cultured with expanded human NK cells (47). This immunosuppressive effect is likely mediated by the increased expression of HLA class I molecules, particularly HLA-B and HLA-E, which act as inhibitors of NK cell activity. Moreover, both CD4+ and CD8+ T cells exhibited a significant reduction in mitogen-induced proliferation following exposure to these THCs. Additionally, THCs were found to induce overexpression of immune checkpoints, such as PD-1 on CD4^+^ lymphocytes, and both PD-1 and CTLA-4 on CD8^+^ cells (47).

Likewise, the expression of the immune-checkpoint SIGLEC5 and its soluble form, which have been identified as a modulator of the immune-response in context like sepsis (144), were not only significantly higher on colorectal THCs after co-culture (121) but also exhibited higher expression in tumour hybrids than CSCs (47). The reduction on CD4^+^ T cell proliferation in the presence of these THCs was reverted in the presence of inhibitory antibody against SIGLEC5 (121). Although these data are limited, they collectively suggest that these THCs are capable of modulating, and even evading, immune surveillance independently (47). However, THCs may utilise additional mechanisms to evade immune attacks and survive the challenging conditions of the bloodstream.

At the same time, it has been well established that CTCs predominantly migrate through the circulatory system as single cells. However, a number of studies have identified the presence of rare clusters of CTCs in various types of cancer (145, 146). Although these clusters are found at significantly lower frequencies than single CTCs, they are considerably more effective in seeding secondary sites and initiating metastatic tumour growth, with over a 100-fold increase in metastatic formation observed in breast cancer models (147, 148). Several investigations further suggest that CTC clustering with TAMs promotes immune evasion and enhances the dissemination and metastatic potential of CTCs (149–151).In spite of all the data discussed above, no reports to date have documented the migration of pure THCs clusters within the bloodstream of patients. Nonetheless, cancer-associated macrophage-like cells (CAMLs), which may also be identified as hybrids due to their multinucleated structure and expression of CD14^+^, CD45^+^, cytokeratin^+^, and EpCAM^+^ markers, have been observed to cluster with CTCs at primary tumour sites (**Figure 2**).

These clusters then enter the vasculature together, migrating to distant organs and acting as potential “metastatic seeds.” CAMLs appear to benefit from the anoikis-suppressing signals provided by CTCs, as well as the downregulation of MHC class II antigen presentation genes in CTCs, which aids hybrids in evading immune surveillance during migration. This symbiotic relationship between hybrids and CTCs suggests that, even if hybrids are not the sole initiating factor in metastasis, they undoubtedly play a pivotal role in the metastatic process (146).

Once circulating cancer cells have reached the secondary niches, they are able to colonize, proliferate and form metastatic tumours in these alternative sites (**Figure 2**). The precise mechanism by which the primary tumour interacts with target organs to establish the pre-metastatic niche remains unknown. However, previous studies have suggested that increased expression of inflammatory chemokines or cytokines induced by tumour-derived growth factors may lead to the establishment of the pre-metastatic niche and promote tumour recurrence (152). Monocytes and macrophages, as the principal agents of tumour fusion, are likely to play a role in the promotion of these pre-metastatic niches. Indeed, some publications demonstrate that macrophages attract myeloid-derived suppressor cells (MDSCs) to form the pre-metastatic niche by increasing CCL12 expression via CXCL10/TLR4 signalling (153, 154).

Likewise, one of the main requirements for the development of the metastatic mass is the vascularization of the nascent tumour. Evidence of pro-angiogenic capabilities has been found in CAMLs isolated from the peripheral blood of pancreatic and breast cancer patients. The pro-angiogenic marker angiopoietin-1 receptor (TIE-2) and the endothelial marker CD146 were observed to stain positive in CAMLs with a variable intensity. This suggests that this subpopulation, identified as hybrids in several reports, might have a role as cellular triggers of neovascularization within metastatic tumours. Given their association with CTCs they are thought to establish themselves at secondary sites, potentially facilitating the vascular development necessary for tumour progression (70).

Overall, it appears that, in addition to their migratory capability and ability to evade the immune, THCs are also inclined to promote the formation of more highly vascularized metastatic tumours. This enhanced vascularisation likely contributes to increased tumour aggressiveness and growth rates, or at least, plays a crucial role in initiating the process of neovascularisation.

## Tumour hybrid cells, drug resistance and recurrent cancer stem cells

**Figure 3:**
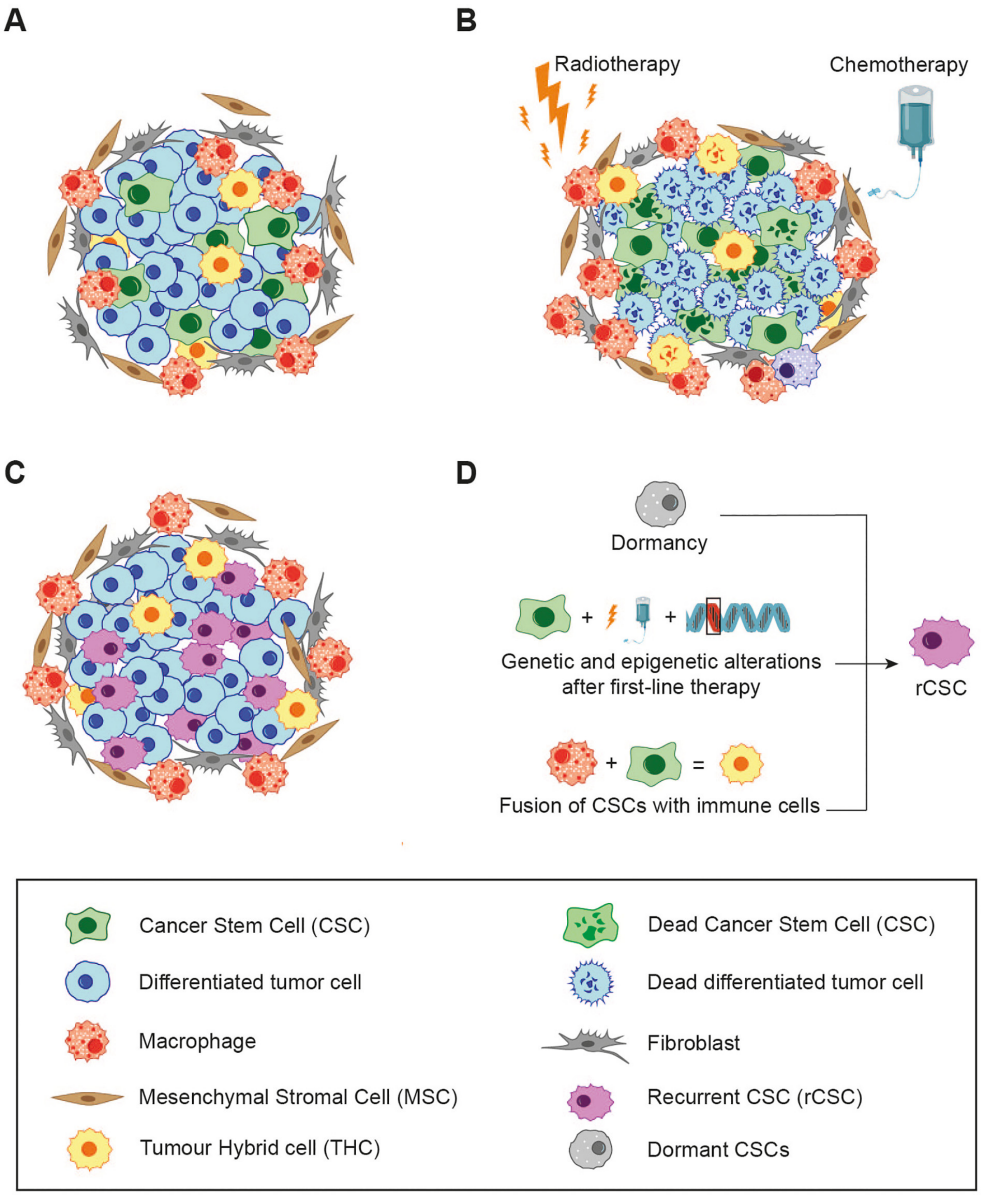
The relationship between cell fusion, cancer therapy and resistance. **(A)** The hierarchy of the primary tumour and the composition of its microenvironment. **(B)** First-line therapies (e.g. chemotherapy/radiotherapy), eliminate the majority of differentiated tumour cells, while a subset of CSCs, which have undergone genetic and epigenetic modification, persists. The resulting cell debris may enter into a state of dormancy or, conversely, release of immunogenic molecules trigger local inflammation, leading to the recruitment of immune cells which could increase cell fusion events. **(C)** All of these events give rise to rCSCs, characterised by enhanced malignancy and resistance to initial line treatments. These rCSCs are responsible for tumour regrow and patient relapse. **(D)** Different mechanism that could explain how CSCs could acquire new properties that give rise to rCSCs.

Current cancer research suggests that tumours are hierarchically structured, much like healthy tissues. This concept is grounded in the belief that tumours consist of a small population of CSCs and THCs, which are thought to arise from normal stem cells or transformed progenitor cells that have acquired self-renewal capabilities through the accumulation of genetic mutations, CSC-derived progenitor cells and a bulk of differentiated tumour cells (**Figure 3A**). These cells along with CSC-derived progenitor cells and a larger population of differentiated tumour cells, collectively maintain the integrity and functionality of cancer tissue. CSCs display several malignant-related traits, including self-renewal, tumour initiation, differentiation capacity, and, more notably, resistance to cytotoxic agents and radiation. This hierarchical organisation of tumours plays a pivotal role in determining the effectiveness of therapeutic interventions (155).

Most anti-cancer treatments, including standard chemotherapy, radiation, hormone therapy, molecular inhibitors, and humanized monoclonal antibodies, are specifically designed to target rapidly proliferating cells. The effectiveness of these therapies is typically measured by two key factors: the rate of tumour reduction and the patient’s disease-free or overall survival. Unfortunately, despite initial positive responses observed in many cancer patients, only a small fraction achieves a definitive cure. This discrepancy underpins what is referred to as “the paradox of response and survival in cancer therapeutics” (156). One hypothesis posits that CSCs may be responsible for cancer recurrences following first-line therapy due to their inherently slow cycling activity and heightened resistance to cytotoxic agents (157, 158). Furthermore, it has been observed that recurrent cancers tend to exhibit increased aggressiveness and resistance to the original treatment, a phenomenon described as “oncogenic resistance” (159–161). These traits of recurrent tumours challenge the original CSC hypothesis, as one would expect that CSCs surviving first-line therapy would produce regrown tumours with similar characteristics, including susceptibility to the same treatment. However, this is often not the case. To explain these inconsistencies, an alternative hypothesis has emerged, proposing the existence of a new subtype of CSCs, referred to as recurrent CSCs (rCSCs) (162).

In line with this definition, rCSCs and their progeny are characterised by increased drug resistance and malignancy. This raises the question of how rCSCs might arise. Several studies support to the notion that chemotherapy induces the selection of genetically altered CSCs, which are subsequently responsible for tumour regrow. This process mirrors the concept of Darwinian evolution, where first line cancer therapies apply selective pressure on the tumour cell population, allowing only the most resistant cells to survive (163). Additional mechanisms may also play a role, for instance the dormancy of CSCs, where a subset of dormant or slowly-cycling CSCs is reactivated by chemotherapy, or enhanced resistance to DNA damage-induced cell death through improved ROS scavenging (164).

On the other hand, cell fusion may represent an alternative mechanism responsible for inducing the genetic alterations in CSCs required for the development of rCSCs. Hybridization has been associated with genetic recombination, aneuploidy, drug resistance, and other malignant traits, as discussed previously. Considering the fusogenic potential of tumour cells, CSCs, immune cells, and BMDCs, it is plausible that fusion events between these populations occur frequently within the tumour microenvironment (**Figure 3B**). This would lead to the formation of a heterogeneous subpopulation of hybrids with distinctive phenotypic characteristics.

Numerous studies have demonstrated the link between cell fusion and acquired resistance to therapies in cancer cells (165–168). For example, Kaur et al., revealed that exposing patient-derived glioblastoma cells to lethal doses of radiation resulted in the emergence of resistant multinucleated giant cells (MNGCs), which originated from fusion events among progenitor cancer cells (169). This supports the notion that cell fusion plays a crucial role in the development of radiation resistance and altered gene expression.

Along these lines, Carloni et al., demonstrated in a metastatic colon cancer model that cell fusion events lead to the emergence of cells with resistance to 5-fluorouracil and oxaliplatin, two of the most widely used pharmacological agents in the treatment of advanced colon carcinoma (165). Although, Aguirre et al., have demonstrated that CSCs, in both lung and colon cancer, are capable to form hybrids with enhanced malignancy (47, 121); there has not been further characterisation of drug and radiation resistance in THCs in this or any other study, to the best of our knowledge.

It is crucial to emphasise that the phenotype of hybrids formed from CSCs and other cell populations in the tumour environment remains unpredictable. Three potential outcomes are possible: (i) the fused cells may exhibit lower malignancy compared to the parental cell lines, (ii) the aggressiveness of the hybrids may be similar to that of the original tumour cells, or (iii) the emerging hybrids could display greater malignancy than the parental populations (170).

These data highlight a significant dilemma in the use of drug therapies due to their dual effects on tumour tissues. At the same time, effective therapies result in the destruction of cancer cell destruction and tumour shrinkage, thereby increasing patient survival.

However, if CSCs survive the first-line treatment, genetic alterations or fusion events triggered by local inflammation may lead to the emergency of rCSCs. These rCSCs then undergo division, causing tumour to regrow with increased malignancy and drug resistance (**Figure 3C**). Thus, it is imperative that CSCs are eradicated to ensure the success of anti-cancer treatments, as this would not only eliminate the primary tumour but will also prevent the formation of rCSCs.

To achieve this therapeutic objective, further research is required on the processes of cell fusion, drug resistance, and the subsequent mechanisms that are implicated in the development of rCSCs (**Figure 3D**).

## Identifying tumour hybrid cells in human *in vivo*

As discussed above, THCs commonly acquire traits such as enhanced migration, immune evasion, and the ability to initiate metastasis. Consequently, it is reasonable to expect that these hybrids may be present in the bloodstream of patients, in addition to being found within primary tumours. The first critical step in identifying them is the determination of specific markers unique to THCs.

CTCs are currently regarded considered as liquid biomarkers for the diagnosis, characterisation of tumour genomic features as well as for monitoring the efficacy of anti-tumour therapies. Their detection in peripheral blood, using the minimally invasive liquid biopsy method via the CellSearch^®^ system, was approved by the FDA several years ago. This system relies on immunoaffinity for specific CTC markers, including a well-defined DAPI^+^ nucleus, cytokeratins 8/18 and/or 19, and EpCAM with the absence of pan-leukocyte marker CD45 expression (115).

However, it is important to note that several subpopulations of cancer-associated cells found in peripheral blood samples from cancer patients do not conform to this definition. This hinders the capacity to detect CTCs *in vivo* (171). Some of them lack EpCAM expression, while others even co-express CD45, along with macrophage/myeloid and tumour markers (172–174). Studies have demonstrated that EpCAM expression may be downregulated during metastatic progression, suggesting that its utility as a predictive biomarker requires careful evaluation (175).

One such population is CAMLs, which have been detected in peripheral blood from patients with breast, pancreatic, NSCLC, renal, oesophageal, and prostate cancer at all stages of the disease, but not in healthy individuals (62, 66). It is noteworthy that CAMLs may even be more prevalent than CTCs in liquid biopsies of cancer patients (70). However, detection rates and isolation methods of CAMLs vary significantly across studies, underscoring for further research into the presence of hybrids in circulation and the establishment of their potential as approved biomarkers.

Another cancer-related subpopulation described to be found in blood circulation of numerous human cancers are THCs. As it can be inferred, THCs are identified by the expression of hematopoietic markers, including CD14, CD45 and CD163, provided by the myeloid partner, as well as common cancer-related markers (e.g. cytokeratin or EpCAM). In fact, some studies suggest that these double-positive cells are more frequent than CTCs in the different tumour types analysed (172, 176).

Along these lines, Ramakrishnan et al., identified THCs in the ascitic fluid of patients with epithelial ovarian carcinoma by utilising a combination of CA125/CD45 and EpCAM/CD45 biomarkers (72), while Aguirre et al., used the CD36/CD14/PANK (pan-cytokeratin) signature to identify hybrids in both tumour tissue and peripheral blood of lung cancer patients (47). Interesting, the first revealing finding was that THCs were not found in healthy controls nor patient with inflammatory pathology such as sepsis. Additionally, patients with the highest levels of hybrids –upper half above the median of circulating THCs— showed a significant correlation with both the size of the primary tumour and the presence of regional lymph nodes metastasis. The same authors in a secondary cohort of 28 lung cancer patients, discovered that the percentage of circulating THCs and gender of the patients could be significant predictors of metastasis (43). In addition, these cells monitored tumour progression and classify the tumour stage (TNM classification) through a minimally invasive blood sample (47).

One the advantages of co-staining methods, such as flow cytometry or immunohistochemistry/immunofluorescence, which simultaneously detect the presence of hematopoietic and cancer-specific markers within the same cell, is the availability of appropriate negative controls.

In this case, macrophages and tumour cells will be positive for only expresses one of these markers, providing clear differentiation. However, as previously noted, a degree of uncertainty relying solely on co-staining to confirm the fusion events, as haematopoietic markers expression by cancer could also result from their inherent genomic instability.

Another shortcoming in the detection of THCs using these methods is that fusion the resulting hybrids may not exhibit any phenotypic differences from with their parental BMDCs or tumour cells, This renders them indistinguishable from non-fused progenitors, often referred to as “dark matter hybrids” (29). Consequently, the reliable detection of THCs in cancer patients remains a significant challenge, with co-staining techniques still considered the gold standard despite their limitations. This barrier is more easily to address in animal models, where a wide range of techniques can be employed to confirm the hybridisation events.

One effective approach involves the use of genetically modified cell lines and mouse models that express fluorescent reporters, such as GFP, RFP or YFP. The advantage of this method is that, rather than relying on cell surface markers, whose expression can be influenced by various factors, fusion is confirmed through the co-localization of specific fluorescent proteins within the same cell. These reporters are exclusively derived from genetically modified populations involved in the fusion process (36, 49, 177).

Another option for identifying hybridisation *in vivo* is HSC or bone marrow transplantation. In addition to being a widely used technique in animal models, this strategy is particularly insightful for studying fusion phenomenon in humans, offering a more reliable alternative to traditional co-staining methods used to detect macrophage-cancer cell fusion (34, 36, 55, 56).

Considering all of the aforementioned, it is crucial for the cell fusion theory to develop reliable methods for detecting THCs *in vivo*. This will be instrumental in elucidating the role these cells play in the initiation and progression of metastasis.

## Strengths and weaknesses of the cell fusion theory of metastasis: a critical discussion

The Cell Fusion theory of metastasis has emerged as a supplementary explanation for various aspects of cancer progression, providing new perspectives on how metastatic traits can rapidly develop within tumours. However, like any theoretical framework, it has its strengths and weaknesses, each requiring careful consideration to determine its overall validity and utility within the broader understanding of cancer biology.

### Strengths of the cell fusion theory

One of the primary strengths of the theory lies in its ability to explain the rapid acquisition of metastatic traits (47, 159, 165, 178). By suggesting that tumour cells fuse with other cells, including immune cells or mesenchymal stem cells, the theory provides a mechanism through which cancer cells can swiftly gain properties such as enhanced migratory capacity and drug resistance. This bypasses the slower, stepwise accumulation of genetic mutations typically associated with cancer progression, offering an alternative pathway for the development of aggressive cancer phenotypes.

The theory also effectively accounts for tumour heterogeneity, a hallmark of many cancers (36, 179, 180). Tumours are often characterised by a wide range of cellular phenotypes, and the fusion of different cell types could create hybrid cells with a blend of characteristics inherited from both parent cells. This diversity could explain the varying levels of malignancy, drug resistance, and metastatic potential within a single tumour, contributing to its ability to evade treatments and adapt to changing environments.

Another compelling aspect of the theory is its ability to provide insight into metastatic organotropism (7, 181, 182). Fusion between cancer cells, especially CSC, and specific immune cells, for instance, may help to explain why certain cancers tend to metastasize preferentially to particular organs. This interaction could help cancer cells adapt to the microenvironments of secondary sites, making it easier for them to establish and proliferate once they have migrated from the primary tumour.

The Cell Fusion theory also offers an alternative explanation for the EMT (21, 22, 25). Fusion between cancer cells and bone marrow-derived cells could result in hybrid cells expressing mesenchymal traits, thereby explaining the transition of epithelial cells into a more migratory, invasive state that is key to metastasis. This offers a novel perspective on the EMT process, complementing traditional genetic and epigenetic explanations.

A further strength of the theory is the breadth of supporting evidence from *in vitro* and *in vivo* studies. Multiple experiments have demonstrated the occurrence of cell fusion events between cancer cells and various cell types, including endothelial cells, macrophages, and mesenchymal stem cells. These findings lend credence to the idea that cell fusion plays a significant role in cancer progression and metastasis.

Finally, it is important to highlight recent lines of investigation into the role of mitochondrial transfer and cell fusion. It has been shown that adenoid cystic carcinoma cells with mitochondrial dysfunction can be rescued by fibroblasts that fuse with them via metabolic revitalisation. This cell fusion increased cancer malignancy and promoted EMT (183).

### Weaknesses of the cell fusion theory

Despite these strengths, the Cell Fusion theory is not without its limitations. One of the most significant challenges to the theory is that studies report fusion occurring in only a small fraction of tumour cells, principally solid tumours, raising questions about the overall importance of this process in driving cancer progression. This low incidence suggests that fusion may not be a major player in all types of cancer, limiting the theory’s universality.

As with conventional cell-based classification and detection methods for CTCs, such as the CellSearch^®^ system, the detection of hybrid cells *in vivo* remains a significant challenge. Over time, hybrids may lose key markers that indicate a fusion event has occurred due to genomic instability, making them indistinguishable from non-fused tumour cells. This “dark matter hypothesis” complicates efforts to identify and study these cells, potentially undermining the theory’s broader applicability.

While genomic instability is a well-known hallmark of cancer, the unpredictable nature of hybrid cell genomic instability also presents a problem. Cell fusion can result in nonviable hybrids due to chromosomal imbalances, and the chaotic nature of chromosomal segregation in these cells makes it difficult to predict their ultimate fate.

Thus, while fusion can generate highly malignant hybrids, it can also produce cells that do not survive, adding an element of unpredictability to the process.

Furthermore, cell fusion has not been observed consistently across all cancer types, which weakens the theory’s capacity to explain metastasis universally. For instance, in some tumour models, cell fusion events are conspicuously absent yet, suggesting that other mechanisms, such as genetic mutations or epigenetic changes, may play a more dominant role in those cases.

The complexity of hybrid cell fate further complicates the theory. After fusion, the fate of hybrid cells is highly variable, with chromosomal segregation occurring in an unpredictable manner. Moreover, it is also important to bear in mind that once a hybrid cell is formed, it could interact with immune and non-immune cells in the TME. These interactions and their randomness make it difficult to establish a clear model for how these hybrids contribute to metastasis or resistance to therapies, leaving a gap in our understanding.

Additionally, many alternative explanations for observed phenomena exist. In this regard, the presence of haematopoietic markers on cancer cells could result from epigenetic changes rather than cell fusion. Similarly, some features attributed to fusion, such as tumour heterogeneity, may also be explained by clonal evolution or mutations.

In terms of research validation, challenges in verifying the theory *in vivo* persist. While *in vitro* studies provide substantial support, replicating these findings in human cancers has been difficult, limiting the direct clinical applicability of the theory.

Finally, the theory’s focus on fusion may result in an overemphasis on fusion events as the driving force behind metastasis, potentially overshadowing other well-established mechanisms such as clonal evolution or genetic mutations. Moreover, while the Cell Fusion theory offers intriguing theoretical insights, it has not yet resulted in the development of targeted therapeutic strategies for metastasis, limiting its practical utility.

Given the factors outlined above, the cell fusion theory cannot be considered the sole explanation for the mechanisms driving the metastatic process in cancer. Based on these weaknesses, in **Table 3** we summarise a decalogue of hot spots that should be addressed to solidify the foundations of this interesting theory.

**Table 3:** Decalogue of hot spots that should be addressed to solidify the foundations of the Cancer Cell Fusion theory.

Critical Points to Investigate	Description
Frequency of cell fusion events	Determine the prevalence of THCs across various cancer types and stages to establish whether cell fusion is a common phenomenon or limited to specific cancers
Metastatic potential	Conduct comparative studies to assess the metastatic capacity of THCs versus non-fused cancer cells to confirm enhanced metastatic properties
Temporal dynamics	Investigate the timing of fusion events throughout cancer progression to understand if metastatic THCs can originate at any stage of tumour development
Molecular mechanisms	Elucidate the specific molecular pathways and signalling cascades involved in cell fusion, hybridization, and subsequent phenotypic changes
Genomic instability	Analyse the genomic alterations and chromosomal rearrangements in THCs to understand how fusion events contribute to increased genomic instability and tumour heterogeneity
Therapy resistance	Evaluate the drug resistance profiles of THCs compared to non-fused cancer cells to confirm their role in treatment resistance
Circulating hybrid cells	Develop methods to detect and characterize circulating THCs and assess their ability to form metastases at distant sites
Organotropism	Investigate the mechanisms by which THCs may acquire specific organ tropism for metastasis, considering factors such as inherited properties from fusion partners
Immune evasion	Examine how fusion with immune cells, particularly macrophages, contributes to immune evasion and survival of cancer cells during the metastatic process
Long-term fate	Track the long-term survival and evolution of THCs in primary tumours and metastatic sites to understand their role in sustained tumour growth and progression
Metabolic reprogramming	Evaluate if the increased aggressiveness of THCs may result from an altered metabolic stress

In conclusion, the Cancer Cell Fusion theory offers valuable insights into certain aspects of cancer progression, particularly regarding rapid metastatic trait acquisition and tumour heterogeneity. While there remains much to be uncovered about fusion events in tumours, further research in this area could provide critical insights and pave the way for future advances in cancer treatment and patient care. Extensive studies are required to dispel the uncertainties surrounding tumour fusion and to illuminate new paths for improving the prognosis of cancer patients.
